# Intravesicular Onabotulinumtoxin A Hemorrhage on Rivaroxaban

**DOI:** 10.1155/2019/5947153

**Published:** 2019-01-02

**Authors:** Allison Eubanks, Katherine Dengler, Daniel Gruber

**Affiliations:** Department of Urogynecology in Obstetrics & Gynecology, Walter Reed National Military Medical Center, Bethesda, MD, USA

## Abstract

Overactive bladder (OAB) is urgency, with or without urgency incontinence. For OAB, an injection of onabotulinumtoxin A (BOTOX®) can be a low-risk outpatient procedure. We present a patient on a novel anticoagulant that experienced excessive bleeding after this procedure. This 80-year-old G2P2002 Caucasian female had a history of urge urinary incontinence. She presented for intravesicular onabotulinumtoxin A injection (150 units) after recent initiation of rivaroxaban (Xarelto®) for her atrial fibrillation. Several hours after an uncomplicated procedure, she presented with anuria and pain after gross hematuria earlier in the day. Her pain was immediately alleviated with bladder irrigation. She was discharged home and remained asymptomatic. With the popularity of the novel anticoagulants, new guidance on management of these medications during procedures is limited. When managing a patient on a novel anticoagulant before any procedure, even a low risk procedure, several factors should be considered to determine if the medication should be held, bridged, or continued. In sum, each patient on anticoagulation undergoing any procedure should be assessed individually for thrombotic risk, bleeding risk, and the procedural risk to best avoid postprocedural hemorrhage.

## 1. Introduction

The International Continence Society defines overactive bladder (OAB) as “urgency, with or without urgency incontinence usually with increased daytime frequency and nocturia” [1]. OAB often has a significant impact on quality of life.

Behavioral modifications are first-line treatment for OAB. The patient should focus on lifestyle changes, such as weight loss, caffeine and alcohol reduction, bladder control strategies, and fluid management. In addition, pelvic floor physical therapy can help with bladder retraining and pelvic floor muscle training. If these conservative measures fail then medication, nerve stimulation, and injection of bladder onabotulinumtoxin A (BOTOX®) can help relax detrusor muscles.

Intravesicular onabotulinumtoxin A injection is a quick, outpatient procedure that has been shown to be more effective than mirabegron in a large meta-analysis and, furthermore, without the invasiveness of sacral neuromodulation as shown in a randomized control trial [1, 2]. The risks of this procedure include urinary tract infections, elevated postvoid residual volumes requiring intermittent catheterization, and hematuria that can occur in 3.6-5.2% of patients [2, 3].

As the likelihood of overactive bladder increases with patient age along with other medical comorbidities, it is not uncommon for patients with OAB to be on blood thinners. Furthermore, patients are on newer, novel anticoagulants that do not have reversal agents. Given the small size of the needle and minimal bleeding expected, there is currently no guidance on stopping or bridging any anticoagulation medications for this same day procedure typically performed in the office setting.

## 2. Case

This is the case of an 80-year-old G2P2002 Caucasian female with a long history of urge urinary incontinence. She presented to clinic for intravesicular onabotulinumtoxin A injection (150 units). She had undergone this procedure seven times with six- to twelve-month intervals, depending on the return of symptoms, ranging from 50 to 150 units of onabotulinumtoxin A since March of 2011. These treatments have significantly improved her symptoms of urgency incontinence after previously trying several anticholinergic medications and sacral neuromodulation.

Her other past medical history is significant for hypertension, peripheral vascular disease, scoliosis, hypothyroidism, diverticulosis, and open-angle glaucoma. She was diagnosed with paroxysmal atrial fibrillation in the beginning of 2016 and developed renal emboli prior to initiation of warfarin. She was transitioned to rivaroxaban (Xarelto®) in mid-2016 after struggling with frequent clinic visits and limited diet while on warfarin. She tolerated this medication transition well.

**Figure 1:**
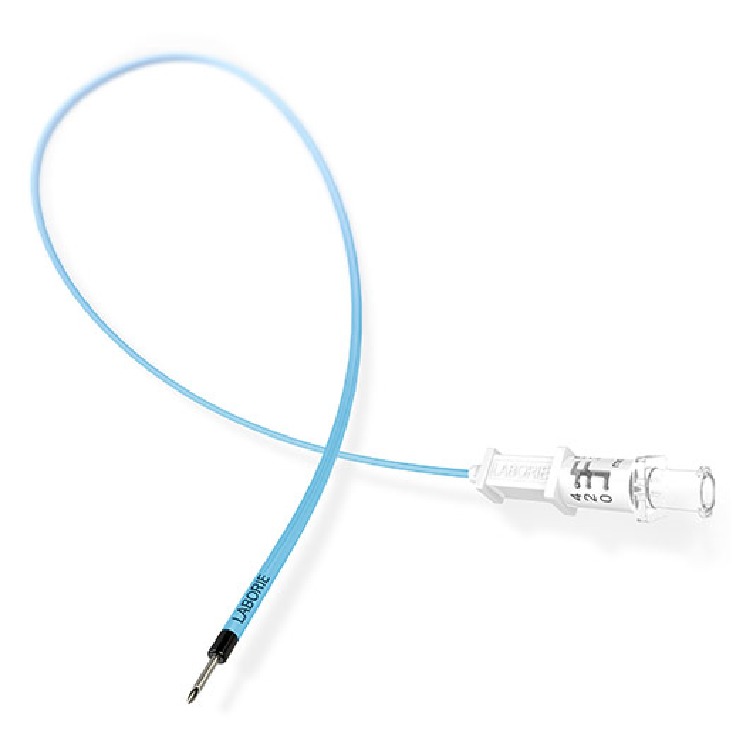
Laborie injeTAK® (Williston, VT) needle.

**Figure 2:**
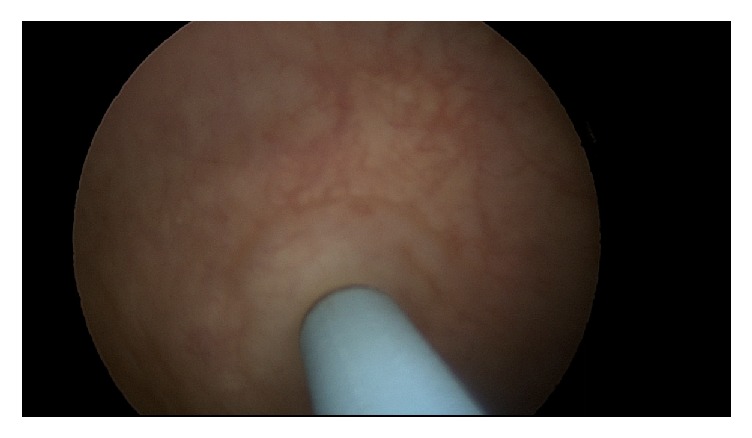
Injection of needle through uroepithelium.

**Figure 3:**
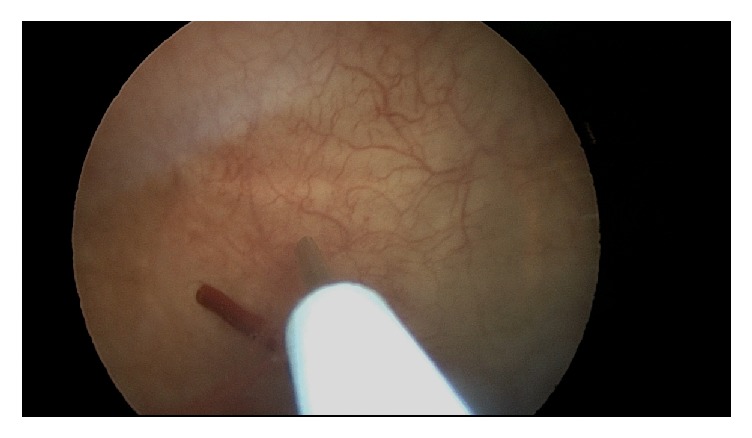
Needle retracting from uroepithelium, appropriate amount of bleeding noted.

**Figure 4:**
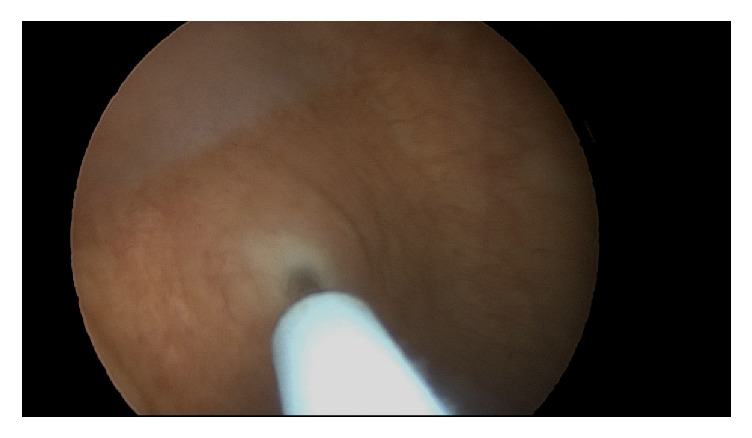
Needle replaced into uroepithelium.

**Figure 5:**
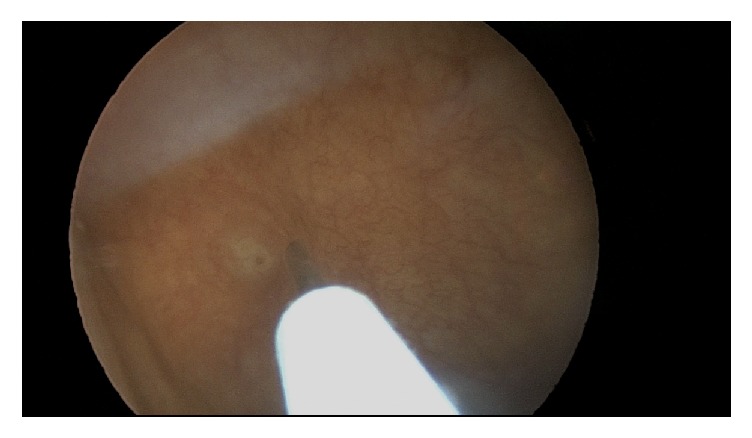
Needle removed completely with no bleeding noted from injection site.

**Figure 6:**
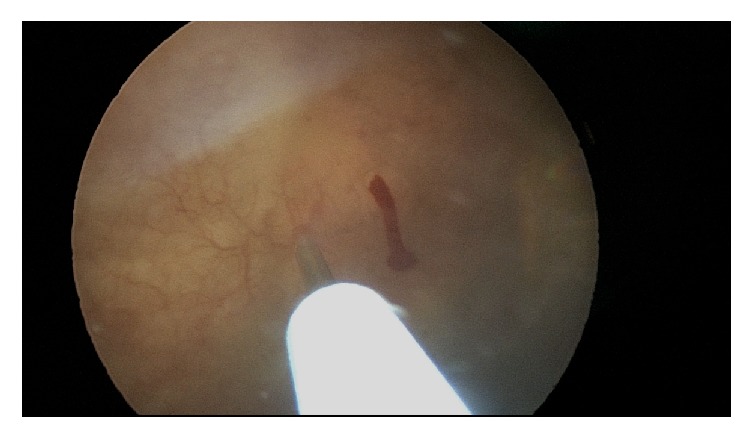
Needle replaced into uroepithelium, appropriate amount of bleeding noted.

The patient had not undergone intravesicular onabotulinumtoxin A injections while on warfarin but however did have a single treatment just one month after initiating rivaroxaban without issues. She returned for the repeat procedure one year later. Using sterile technique, the bladder was filled with 20 mL of 1% lidocaine and 2% viscous lidocaine was administered to the urethra 15 minutes before the procedure. The onabotulinumtoxin A dose was reconstituted in 20 milliliters' saline solution. A 30-degree operative cystoscope was inserted into the bladder. A total of 150 units of onabotulinumtoxin A were injected into the bladder wall in 20 sites with one milliliter in each injection covering the posterior surface of the bladder wall and sparing the trigone. The depth of the injection was set at 3 mm under the urothelium layer with the Laborie injeTAK® (Williston, VT) needle; see Figures 1–6. The patient tolerated the procedure well. There was minor bleeding from a few needle injection sites, but not an atypical amount. She voided immediately after the procedure without difficulty and was sent home from the office feeling well.

Seven hours later, the patient called the clinic and reported two hours of frank blood in her urine with passage of several clots. She reported drinking large volumes of water and frequent voiding without improvement. She was encouraged to keep her bladder full to allow distension to tamponade the bleeding, rather than empty frequently. She was given strict return precautions to present to the emergency department if her bleeding persisted or for any retention symptoms. The following morning, the patient presented to the emergency department in pain with no urine output for the last several hours. She was given intravenous morphine, which alleviated her immediate pain.

A 14-French Foley catheter was placed and slowly drained dark red urine with clots. Only about 200 milliliters returned in the catheter. Several flushes were attempted to alleviate the blockage without success. Continuous bladder irrigation (CBI) was initiated which cleared the obstruction. The patient continued to put out frank blood with the CBI. Her complete blood cell count was normal and did not show a significant drop [15.2/45.8 to 14.0/43.5]. Her pain was completely alleviated with the CBI until new clots developed that caused a repeat obstruction two more times overnight requiring Foley adjustment.

Cardiology was consulted to aid with management of her anticoagulation in the setting of bleeding and her rivaroxaban was held for three days. Following 36 hours of CBI, her urine was lighter, indicating bleeding cessation. The CBI was stopped and the patient was taught to flush the catheter with normal saline.

The patient returned for a scheduled clinic appointment four days later and reported that she only had a few episodes of hematuria that resolved with minimal flushing. The bladder was back-filled with 400 milliliters and the Foley catheter removed. She voided 350 milliliters of clear yellow urine without difficulty. The patient continued on prophylactic nitrofurantoin for ten days. She followed up three weeks later without return of the hematuria. She reported some continued overactive bladder symptoms such as frequent voiding, some urgency incontinence upon standing, and nighttime voids up to four to five times per night; however the frequency of these symptoms was all decreased from prior to her procedure. She continues to find the best solution to manage her symptoms.

## 3. Discussion

Despite the resulting hemorrhage from this intravesicular onabotulinumtoxin A injection, bleeding with this procedure is rare. This case is significant in that bleeding is so rare and the injection sites are so small. The patient had additionally not bled during the procedure while previously on rivaroxaban, approximately one year priorly. She reported no tobacco or alcohol use, and her medications at the time of this procedure had not changed.

Antifactor Xa was not collected during or around the time of either procedure. Her weight and renal function remained unchanged and liver function was previously normal. However, it is important to consider any changes to these values in patients on novel anticoagulants as they can cause patients to be supratherapeutic and increase risk of bleeding with even minor procedures. There is only one case report that reviews the management of a patient using warfarin before intravesicular onabotulinumtoxin A injection [4]. The purpose of that publication was to simply demonstrate the proper discontinuation, bridging management, and reinitiation of warfarin to safely complete the procedure for a patient [4].

Allergan, the onabotulinumtoxin A manufacturer, does not list anticoagulation as a contraindication and suggests only managing patients on anticoagulant therapy appropriately to decrease the risk of bleeding [5]. It is at the clinician's discretion to determine the procedural bleeding risk and weigh the patient's thrombotic risk.

Spyropoulos et al. and the American College of Cardiology (ACC) both compiled a list of procedures and delineated their bleed risk level [6, 7]. According to the list from Spyropoulos et al., a bladder biopsy is considered a low procedural risk, with a 2-day risk of major bleed at 0% to 2%. According to the list from the ACC, as contributed by Dr. Bruce Jacobs and the American Urological Association, uncomplicated ureteral stenting and exchange is considered a low bleed risk. Intravesicular onabotulinumtoxin A injections are much less invasive than bladder biopsies and less invasive than ureteral stenting. ACCP gives 2C recommendations regarding continuing vitamin K antagonists (VKAs) in minor dermatologic procedures and cataract surgery. It is also recommended to discontinue VKAs for two to three days before the procedure or coadministration of an oral prohemostatic, a suggestion that may be applied to this situation [8].

The patient was taking rivaroxaban for paroxysmal atrial fibrillation, diagnosed in the wake of a renal infarct attributed to a cardioembolic source. Given the prior documented history of cardioembolic phenomenon to the kidneys, the patient's cardiologist determined that she requires lifelong anticoagulation. Her CHA_2_DS_2_-Vasc score of at least five points estimated her stroke risk at 7.2% per year. It was not unreasonable to continue anticoagulation during this minimal procedure for such a high-risk patient.

In future intravesicular onabotulinumtoxin A injections performed for this patient, and similar patients, it would be reasonable to consider discontinuing her anticoagulant for two to three days before procedure and bridging with low-molecular weight heparin or heparin. The physician could also consider coadministering an oral prohemostatic agent such as tranexamic acid. Working with the prescribing physician managing these medications and comorbidities may prove beneficial. However, for other intravesicular onabotulinumtoxin A procedures performed for OAB, we would continue to treat this as a low-risk procedure. Each patient on anticoagulation undergoing any procedure should be assessed individually for thrombotic risk, bleeding risk, and the procedural risk to best avoid postprocedural hemorrhage.
